# Genetic modification and genetic determinism

**DOI:** 10.1186/1747-5341-1-9

**Published:** 2006-06-26

**Authors:** David B Resnik, Daniel B Vorhaus

**Affiliations:** 1 Bioethicist and IRB Vice Chair, NIEHS/NIH, Box 12233, Mail Drop NH06, Research Triangle Park, NC, 27709, USA; 2 Harvard Law School, 101 Madera Lane, Chapel Hill, NC 27517, USA

## Abstract

In this article we examine four objections to the genetic modification of human beings: the freedom argument, the giftedness argument, the authenticity argument, and the uniqueness argument. We then demonstrate that each of these arguments against genetic modification assumes a strong version of genetic determinism. Since these strong deterministic assumptions are false, the arguments against genetic modification, which assume and depend upon these assumptions, are therefore unsound. Serious discussion of the morality of genetic modification, and the development of sound science policy, should be driven by arguments that address the actual consequences of genetic modification for individuals and society, not by ones propped up by false or misleading biological assumptions.

## Background

In *Brave New World*,[1] Aldous Huxley imagined a society in which the government manufactures five different human castes designed to perform different roles. Four decades after the publication of that dystopia, Robert Nozick[2] developed another futuristic scenario, the genetic supermarket, to prompt discussion of the moral implications of eugenics conducted not by the state, but at the level of individuals. In the genetic supermarket, as Nozick portrays it, becoming a parent is like buying a new car. If you want to have a child that will be male, athletic, musically gifted, heterosexual, 6'1" tall, with brown hair, blue eyes, and an IQ of 140, then you simply purchase the goods and services necessary to create that exact child. Parents can design children to fulfill their own desires, hopes, and aspirations. Since the 1970s, numerous authors have examined the moral implications of "designer babies," and popular films, such as Blade Runner, GATACCA, and X-Men, have also explored the subject. And fully five different presidential committees have dealt with ethical issues raised by the genetic modification of human beings [3-7].

As a libertarian, Nozick defended a *laissez-faire *approach to genetic modification, arguing that the government should not interfere with the market forces that influence procreation. Other writers have put forth similarly vigorous defenses of reproductive freedom [8,9]. Many commentators, however, have argued for government regulation of genetic modification in order to protect important values, such as social justice and the welfare of unborn children [10-12]. Finally, some have argued that genetic modification should be banned, since any attempt to modify the human genome violates human freedom and dignity, and leads us down a perilous path toward social, political and biological disaster [13-18].

Since the risks to unborn children from genetic engineering mistakes are not currently known, and are likely substantial, few authors support the no-regulation view with regard to modifying the human genome. Most of the current debate is between those who think that genetic modification should proceed under some type of regulatory scheme, and those who think that the best solution is to ban genetic modification entirely [19]. Those who favor regulation see nothing inherently wrong with genetic modification: the morality of genetic modification depends on an adequate understanding and evaluation of the medical, social, economic, political, and biological consequences. Society should take appropriate steps to control genetic modification in order to maximize its benefits and minimize its harms [11,12]. Those who favor a ban, however, believe there is something inherently wrong with genetic modification, that there are inevitable, unavoidable, and undesirable consequences associated with modifying the human genome [16].

In this article, we examine four arguments used to support the view that there is something inherently wrong with genetic modification. These arguments aim to pre-empt analysis of actual or expected medical, social, economic, political, and biological consequences, and to argue for a comprehensive ban of the technology due to its very nature. We demonstrate that these arguments against genetic modification – the freedom argument, the giftedness argument, the authenticity argument, and the uniqueness argument – all necessarily assume a strong version of genetic determinism. If these deterministic assumptions are false, as we maintain they clearly are, then these particular arguments against genetic modification lose their logical force. Thus, serious discussion of the morality of genetic modification is more properly focused on arguments that examine and address the expected consequences of genetic modification for individuals and society, and not on ones that would pre-empt such a discussion by arguing that genetic modification is inherently objectionable.

Our analysis is divided into three separate parts. First, we attempt to define two important terms: "genetic modification" and "genetic determinism." Second, after explaining the difference between the stronger and weaker forms of genetic determinism, and examining why the stronger versions of genetic determinism appear very rarely in biology, we unpack four common arguments against the use of genetic modification and show how they lean heavily on assumptions of strong genetic determinism. Finally, we argue that moral assessments of genetic modification should consider arguments that pragmatically examine the biological, medical, social, and economic consequences of genetic modification, rather than those that rely on scientifically unwarranted assumptions of genetic determinism to portray genetic modification as inherently objectionable.

### What is genetic modification?

"Genetic modification" has many apparent synonyms in the literature: genetic engineering, genetic enhancement, germline engineering, germline enhancement, germline therapy, germline manipulation, genome manipulation, and so forth. In this paper, when we speak of "genetic modification" we mean the process of intentionally altering human genes for the purpose of producing offspring with those genetic changes [20]. We use the term "genetic modification" because it covers a wider range of cases than other terms, and because it does not assume a distinction between genetic therapy and genetic enhancement, one which is difficult to maintain and which may not be as morally significant as is often assumed [20,21]. Some examples of genetic modification include:

• Insertion, deletion or transposition of genes or DNA sequences in human gametes, human zygotes or early embryos;

• Transfer of ooplasm or nuclei in human zygotes;

• Introduction of artificial chromosomes in human gametes or zygotes.

Some examples of procedures that we do not consider to be genetic modification include:

• Insertion or deletion of genes or DNA sequences in human somatic tissues;

• Insertion, deletion, or transposition of genes or DNA sequences in human gametes, zygotes, or embryos for research purposes, with no intention of creating human offspring;

• Transfer of ooplasm or nuclei for research purposes only in human zygotes;

• Introduction of artificial chromosomes in human gametes or zygotes for research purposes only;

• Pre-implantation diagnosis and embryo selection;

• Prenatal testing and selective abortion.

The *sine qua non *of genetic modification is permanent genetic alteration: the intentional production of human offspring with artificially induced genetic changes, or "designer babies."

### What is genetic determinism?

"Genetic determinism" is another term that needs clarification. In philosophy, determinism is usually equated with the problem of free will: We are compelled to make the choices that we make as a result of previous circumstances, and we cannot make choices that are genuinely free. This type of determinism, which we shall call psychological determinism, has some profound implications for morality and the law, since we normally ascribe moral or legal responsibility to people under the assumption that they can choose freely. Over the years, philosophers have developed three basic positions on the problem of free will: 1) hard determinism, which holds that we cannot make free choices; 2) indeterminism, which holds that human actions result from spontaneous acts of the will that break free from the world's causal nexus, and 3) compatiblism, which holds that free will is compatible with determinism [22]. According to some compatibilists, actions may be considered "free" if they are caused in the appropriate way. For example, a "free" act is one that results from reasoning and deliberation rather than external forces or emotional compulsions [22].

While questions about the metaphysics of human freedom are of the utmost importance in philosophy, they are not the focus of this article. However, there are some important parallels between psychological determinism and genetic determinism, since the interpretation of causation plays a pivotal role in both of these doctrines. Also, as we shall see below, worries about genetic determinism can reinforce concerns about psychological determinism [23]. Since the concept of causation plays a central role in various forms of determinism in philosophy and science, we will say a bit more about causation. We do not have space in this paper to provide a detailed analysis of causation, but we will make a few critical points that are relevant to questions about genetic determinism (for further discussion of causation, see Salmon, 1997; Tooley, 2000) [24,25].

First, causation is a temporally ordered relationship between events, properties, or processes. In the statement, "lightning caused the forest fire," lightning precedes the forest fire. Second, almost all causal relationships involve more than one factor (or condition). For example, the dryness of the forest and wind velocity would also be causal factors in the forest fire. Very often, causal factors serve as background assumptions in causal explanations [25]. For example, a person who claims that lightning caused the forest fire would be assuming that there was enough oxygen in the atmosphere to fuel the fire. Third, causal statements can be used in explanation or prediction [24]. For example, the statement "smoking causes lung cancer" can be used to predict that a person who is a heavy smoker will develop cancer, or to explain why a heavy smoker develops cancer.

Fourth, and of greatest import for our purposes, causal relationships can be either deterministic or probabilistic [24]. For example, consider the claim "If you drop a rock, it will fall". Many would consider this to be a deterministic form of causation because it does not make a reference to the probability, or chance, of an event occurring: the rock *will *fall if it is dropped (assuming background conditions, e.g. there is not a strong wind pushing the rock up). However, consider research on smoking and lung cancer. Smoking causes lung cancer, even though many smokers do not develop lung cancer [26]. If you smoke, you may not get lung cancer, but smoking increases your probability of getting lung cancer. While deterministic causation is common in the physical sciences, it is very rare in biology and in medicine. Most explanations and predictions in the biomedical sciences are probabilistic, not deterministic [27]. As we shall soon see, despite assumptions to the contrary, most of the causal claims related to genetic determinism are probabilistic, not deterministic.

With the preceding comments in mind, we now consider genetic determinism. Genetic determinism can be loosely defined as the view that genes (genotypes) cause traits (phenotypes) [28]. This definition is almost trivially true, because most traits have some type of genetic basis. More precisely, one could say that trait T is genetically determined if it is caused by gene G. For example, a person who is born with two copies of the Sickle Cell allele will almost certainly develop Sickle Cell Disease (SCD), provided that some necessary environmental conditions obtain. SCD is said to be genetically determined. However, even this definition is not precise enough, since it ignores that fact that genetic causation is usually not deterministic in the strict sense: genes often merely increase the probability, though sometimes quite substantially, that an organism will develop a particular trait [29,28]. For example, BRCA1 and BRCA2 mutations increase a woman's lifetime probability of developing breast cancer from 36% to 85%, compared to 13.2% for the general population [30]. People with the APOE4 mutation have an increased risk of developing Alzheimer's disease, but most of them will not develop this condition [31]. To differentiate between these types of genetic causation, we distinguish between three different forms of genetic determinism:

**Strong genetic determinism**: gene G almost always leads to the development of trait T. (G increases the probability of T and the probability of T, given G, is 95% or greater).

**Moderate genetic determinism**: more often than not G leads to the development of T. (G increases the probability of T and the probability of T, given G is greater than 50%).

**Weak genetic determinism**: G sometimes leads to the development of T. (G increase the probability of T, but the probability of T is still less than 50%.)

Geneticists have a term – "penetrance" – that is similar to what we have in mind here. Penetrance is often defined as the percentage of members of a population that will have a particular phenotype, given a particular genotype [32].

Strong genetic determinism is not very common: the vast majority of traits are either moderately or weakly determined by genetics [33,32]. There are several reasons why strong genetic determinism turns out to be rare. First and foremost, the environment plays a very important role in the expression of most genes. An individual with the genetic potential to be six feet tall will not reach this height if he/she lacks a proper diet during childhood; an individual with a genetic predisposition toward alcoholism will not develop this disease if he/she never drinks alcohol. The complex interaction and interdependence of genes and environments, a fundamental and frequently ignored reality of biology, undermines the notion that genotypes alone determine (or cause) phenotypes [34,35]. Second, most traits are epistatic: they are determined not by a single gene but by many different genes. Dozens or even hundreds of genes may play a causal role in the genesis of complex traits such as intelligence, personality, or athletic ability. So, a single gene may only have a small influence on the development of the trait [32]. Third, development (or epigenesis) has a significant impact on gene expression, i.e. how organisms convert genetic information into traits [36]. Because developmental patterns and processes influence gene expression, two organisms with identical genomes and substantially similar environments may still express different phenotypes [35]. Identical twins, for example, usually look very similar but may possess subtle variations in hair, skin pigmentation, facial shape, fingerprints, or dental impressions. Even among cloned animals there may also be phenotypic differences [37].

Since most traits are not strongly genetically determined, Nozick's genetic supermarket scenario strikes us as having little grounding in reality. While parents may one day select among genes that will increase or decrease the odds that their children will develop specific traits, creating children will not be like shopping for an automobile or designing a home. Despite this reality, popular culture, the media, and politicians are apt to ignore the fact that strong genetic determinism is almost entirely a myth. Journalists continue to speak of "genes for obesity," "genes for alcoholism," and "cancer genes," as if genes exist that, once discovered, will give individuals the ability to simply "shut off" obesity, alcoholism, or cancer with a few simple snips to their genome.

The idea of a "genetic age" continues to exert a powerful influence on popular culture and many individuals regard genes as possessing nearly magical power, as well as moral and religious significance [38]. As explained below, these popularized and sensationalized imaginations of the power of genotypes to control and determine phenotypes have influenced, and indeed serve as the foundation for, some of the most common contemporary arguments employed against genetic modification.

Before concluding this section of our article, we briefly discuss three other points, each of which call into question the deterministic portrayal of genes and are, therefore, relevant to understanding the role of genetic determinism in arguments against genetic modification. First, advances in behavioral genetics – the study of the genetic basis of behavior – suggest that genetic determinism may have implications for psychological determinism [39]. If an individual has a gene linked to a type of behavior, such as aggression, does this gene undermine the individual's free will when it comes to that behavior? Can an individual with a genetic tendency toward aggression choose to not act aggressively? If he/she has a gene that predisposes him to aggression, should he/she be held legally or morally responsible for his actions? Behavioral genetics has led some legal scholars to raise questions like these [39,40]. We will not enter the debate about the implications of behavioral genetics for criminal justice. However, we would like to note that those who are troubled by these questions must assume two different kinds of determinism to get their arguments off the ground: strong genetic determinism *and *psychological determinism. To seriously claim that a gene linked to aggression invalidates the free will of a person with that gene, one would need to show that the gene both strongly determines aggressive tendencies in people and, in addition, that people with these tendencies are not free to act differently. Such a person would truly be a puppet controlled by his/her genes [23].

Our second point distinguishes between determinism and fatalism. Fatalism is the view that specific outcomes or events will occur in our lives no matter what we do. The classic example of fatalism is the myth of Oedipus. A prophet told Oedipus that he would kill his father and marry his mother. To avoid this horrible outcome, Oedipus went to live far away from his homeland, and was still unable to avoid fulfilling the prophecy. Analogously, genetic fatalism is the view that we cannot avoid specific genetically predetermined outcomes, no matter what we do or what happens to us: our fate is in our genes. According to Lewontin[34], genetic fatalism also has social and political implications, because it implies that much of social and political realities are beyond our control.

Although genetic fatalism has also become a popular belief in some circles, critical examination of this idea shows that it does not square with modern biology, or with commonsense. As an almost trivial example, for genetic fatalism to be true an individual possessing a gene responsible for a specific type of cancer must develop that type of cancer, no matter what he or she does. Clearly, this is not the way the world works. Leaving aside any discussion of genetic causation and assuming that, in this case, the gene strongly determines the phenotype (cancer), science might yet discover a pre-emptive cure for that particular cancer and thus prevent phenotypic expression. Or, to offer one macabre alternative, the person might get hit by a bus and die before ever developing the cancer.

Our final point concerns the relationship between determinism and control. As we show below, arguments against genetic modification expound on moral problems that can arise when one attempts to control human traits. However, individual traits, just as events in the world, may remain beyond human control despite being strongly determined. For example, the collision of an asteroid with the Earth is determined by the size, velocity, and orbit of both celestial bodies, along with certain other conditions. However, despite our knowledge of these factors, we will remain unable to prevent such a collision unless and until human ingenuity and technology enable the successful manipulation of these causal factors.

A similar situation is not hard to envision with respect to human biology and genetics: a trait might be strongly genetically determined but, nevertheless, remain beyond our control as the result of either its complexity, including its interactions with the environment, or our own lack of scientific and technological ability. Suppose that intelligence proves strongly genetically determined but that there are over two hundred different separate genes involved in the expression of this trait. The sheer complexity necessarily entailed by hundreds of interrelated genes might, in this hypothetical scenario, hinder and forever frustrate our attempts to control or modify intelligence by way of genetic manipulation. Moreover, since genes often produce more than one phenotypic effect (a condition known as pleiotropy), we may find it difficult to maximize intelligence without simultaneously causing adverse affects, such as anxiety and aggression [41,42].

### Arguments against genetic modification

Since the 1970s, scholars have developed a variety of arguments against genetic modification of human beings. We will not canvass all of these arguments here (for a review, see Resnik, Steinkraus, and Langer [11]; Buchanan, Brock, Daniels, and Wikler [10]; Mehlman [18]), but we will divide them into two basic types: consequentialist and non-consequentialist. Although critics of genetic modification sometimes conflate consequentialist and non-consequentialist concerns, we find it useful to clearly distinguish between these two different forms of argument, since they are structured quite differently [43]. Consider pornography as an example. The non-consequentialist argument, that pornography is inherently wrong, is logically different from the consequentialist argument that pornography is wrong because it produces bad social consequences. The non-consequentialist who believes that pornography is inherently wrong is likely to favor a total ban on pornography, whereas the consequentialist will be willing, at least in certain circumstances, to legalize and to regulate pornography.

Consequentialist arguments assert that the negative consequences of genetic modification far outweigh any benefits that may occur. These may include harms to children and to future generations; loss of biological or cultural diversity; economic costs; and the degradation of social values such as acceptance of disabled people, respect for the value of human life, and equality of opportunity. Non-consequentialist arguments claim that there is something *inherently *wrong with genetic modification of human beings: genetic modification would still be wrong even if the good consequences of modification outweighed the bad. With this distinction clearly in mind we turn now to examine four of the most influential non-consequentialist arguments: the freedom argument, the giftedness argument, the authenticity argument, and the uniqueness argument.

### 1. Genetic modification and human freedom

The freedom argument claims that genetic modification interferes with the ability of the modified human being to make free choices. Hans Jonas[44,15] developed this argument in the early 1970s as an objection to cloning. Leon Kass[14,45], Dena Davis[46], and Francis Fukuyama[17] have developed and expanded different versions and interpretations of this argument. The freedom argument can be understood in three different ways:

(a) Genetic modification prevents that person who has been modified from making free choices related to the modified trait. The modifier controls the person's future by controlling his/her genes. If you have been given a gene for musical talent, you have no choice but to become a musician. We will call this the Puppet Critique.

(b) Genetic modification limits the options of the person who is modified by limiting their range of behaviors and life plans. A person with a gene that causes him/her to grow to a height of seven feet cannot become a jockey. We will call this the Open Future Critique.

(c) Genetic modification interferes with the person's ability to make free choices by increasing parental expectations and demands. A person with a gene for musical talent will face enormous pressure to become a musician. We will call this the Parental Expectations Critique.

As we now show, all three of these critiques, covered by the broader umbrella argument that genetic modification interferes with the freedom of the modified individual, rest heavily upon unsupportable assumptions of genetic determinism.

#### a. The Puppet Critique

The Puppet Critique assumes strong forms of genetic and psychological determinism. For this argument to work, one must assume that the gene strongly determines the development of a particular behavioral trait and that the person will be unable to avoid expressing that trait. The modified person with a gene for musical ability will have no choice but to develop this ability to its fullest extent: he or she will become a professional musician. Kass develops this sort of argument in his critique of human cloning:

The child is given a genotype that has already lived, with full expectation that this blueprint of a past life ought to be controlling of the life that is to come. Cloning is inherently despotic, for it seeks to make one's children (or someone else's children) after one's own image (or an image of one's choosing) and their future according to one's will. In some cases, the despotism may be mild and benevolent. In other cases, it will be mischievous and downright tyrannical. But despotism – the control of another through one's will – it inevitably will be [[45], at pg 24].

This type of argument, the cloner as the despotic puppet-master, is highly problematic because, as previously discussed, it relies on dubious biological and psychological assumptions. First, contrary to Kass's implication, exerting control over a child's genotype does not give one despotic control to shape "their future according to one's will." Both environmental and developmental factors *must *be considered. A person with a gene for musical ability may not develop this ability if he/she loses his/her hearing as a result of childhood illness, is not exposed to music at an appropriate time, or is not afforded the chance to play and to practice an instrument. To seek the sort of despotism that Kass has in mind – "to make one's own children...after one's own image" – will require more than just a reproductive decision; it will require a lifelong commitment.

Even more problematic for the Puppet Critique is the assumption of psychological determinism, as the individual might decide not to pursue a modified trait to its fullest extent. The most genetically gifted musician might nevertheless forgo a career as a musician or a composer, favoring life as an accountant or attorney instead. Indeed, the person might even come to share Kass' dismal opinion of cloning, and rebel against what his/her parents believed was a genetic gift for that or any number of more benign reasons [47]. At its most basic level, the Puppet Critique relies on misstatements of scientific reality, and plays on the public's worst fears about the powers of genetics [23].

#### b. The Open Future Critique

The Open Future Critique also assumes a strong form of genetic determinism (although it does not assume psychological determinism) in arguing that genetic modification narrows the range of life choices available to a modified individual. If life is conceived of as a journey with many different possible roads that an individual might travel then genetic modification, according to this critique, may close off some of those roads. Dena Davis[46] argues that one form of genetic modification violates the child's right to an open future: deaf parents deciding to conceive a child that will have a gene that will make him or her deaf. According to Davis, such decisions close off options for the child, such as the ability to participate fully in hearing culture.

There are, we believe, conceptual difficulties with the right to an open future critique: How can one compare different possible futures? What makes one future more open than another? Isn't it good to close off certain choices? We will set these issues aside and assume that parents have an obligation to avoid making decisions that are likely to narrow the range of arguably desirable choices, or life pathways, that their children might otherwise have available to them.

Even if we grant this contestable point, genetic modification closes off the child's open future only if one assumes a certain strong form of genetic determinism: that by choosing a particular genotype one produces a child with fewer options. Otherwise, in the absence of a strong causal link between genotype and phenotype, genetic modification might not close off any options for the child. Recall that genes may not be expressed (consider, again, environmental and developmental constraints, as well as the general complexity of a given trait). Similarly, due to the reasons outlined above, even if a desired trait is successfully expressed it may not actually restrict options for the child. Of course, we do not deny that there are *some *genotype-phenotype relationships in which genes are strongly determinative. And one can imagine certain scenarios – such as intentionally modifying a child with a gene that is strongly determinative for a serious disease or disability – which would clearly violate the child's right to an open future, and which would be objectionable for a host of other reasons as well. But the open future critique paints with a far broader brush, alleging that the act of modification *per se *impacts the child's right to an open future. And it is this claim that we reject: there are different forms of genetic modification and, correspondingly, they impact a child's right to an open future in different ways. The claim that genetic modification is inherently violative of a child's right to an open future is one that ignores the varying degrees of genetic determinism and it is, therefore, one that we reject.

#### c. The Parental Expectations Critique

The Parental Expectations Critique assumes that parents will burden their genetically modified children with unreasonably high expectations and demands. To continue with our music example, a parent who seeks to have a child with the genes of a musical prodigy will steer the child toward a career in music and, in so doing, will interfere with that child's decision-making.

[A]n enlarged degree of parental control over the genetic endowments of their children cannot fail to alter the parent-child relationship. Selecting against disease merely relieves the parents of the fear of specific ailments afflicting their child; selecting for desired traits inevitably plants specific hopes and expectations as to how their child might excel. More than any child does now, the "better" child may bear the burden of living up to the standards he was "designed" to meet. The oppressive weight of his parents' expectations – resting in this case on what they believe to be undeniable biological facts – may impinge upon the child's freedom to make his own way in the world [[6], at pg 55–56].

The image of overbearing parents, empowered and emboldened by the tools of genetic modification, represents a well-worn critique repackaged using updated language and terminology. To the extent that genetic modification represents a means by which parents seek to impose their expectations on their children it is no different in kind from other assisted reproduction technologies that have been in operation for decades. The parent who seeks to give her child the "music gene" is hardly any different from the parent who seeks a world-class musician to be a sperm or an egg donor, and the expectations placed on the resulting child will not be fundamentally of a different kind simply because genetic modification was used instead of in vitro fertilization. Indeed, some scholars suggest that, paradoxically, parents who go to the trouble to use assisted reproduction may be less likely to place high demands on their children due to factors such as age and emotional maturity [48].

To the extent that the Parental Expectations Critique applies specifically to genetic modification it relies, not surprisingly, on a misstated deterministic relationship between genotype and phenotype. And as we have discussed at length, it is simply not the case that the ability to specify genotype will present parents with the opportunity to control phenotype, no matter how strongly they *expect *to be able to do so. However, in an important respect the Parental Expectations Critique relies merely on the *belief in*, and not the *reality of*, strong genetic determinism. This critique of genetic modification, unlike other freedom-based critiques, need not make the demonstrably unsupportable assumption that strong determinism links most genotype-phenotype pairs; it need only assume that parents act under this belief and expect their children to develop accordingly. Parents acting under this belief may also take steps, wittingly or unwittingly, to control the child's environment in the hope or the expectation that the child will develop specific phenotypes [49].

While it is certainly possible that some parents who utilize genetic modification will naïvely assume, implicitly or explicitly, the presence of a strong genetic determinism, this is a property that is inherent neither in the process of genetic modification nor in the status of being a parent. With adequate counselling and education most parents may be steered away from this problematic assumption. For instance, most fertility specialists aim to ensure that prospective parents receive adequate information about the nature of procedures used in assisted reproduction, potential benefits, limitations, potential risks, and costs. Informed consent is one of the important ethical pillars of reproductive medicine [50]. Appropriate education and counseling should similarly inform parents of the limited ability of genetic modification to control the traits of an offspring. Patients usually understand that a suggested course of treatment does not come with a guarantee of success. And prospective parents recognize that a proposed infertility treatment does not guarantee a pregnancy. With appropriate education and counselling parents will come to similarly understand that genetic modification cannot guarantee a musical genius, a star athlete, or a child that is forever free from cancer. They will learn that except in rare cases, genetic modifications deals in the realm of probability, of increased or decreased likelihoods, and not with the definite causation of specific phenotypic traits.

Thus, to the extent that the this critique is grounded in the fear of increased parental expectations due to a heightened or expanded ability to exert control over the phenotypic development of children, it succumbs to the same genetic determinism counter-arguments that we have raised repeatedly. And to the extent that it depends on the *perception *of genetic determinism it does not support the conclusion that genetic enhancement technologies are inherently problematic and must be banned; only that our understanding of them is imperfect. Either way, the solution is not to reject genetic modification entirely. Rather, it is to continue to work to treat genetic modification seriously, to evaluate the medical, social, economic, political, and biological consequences of specific technologies, and to promote a more accurate and widespread understanding of what genetic modification actually entails, and what it can and cannot accomplish.

#### 2. Genetic modification and giftedness

We group together a number of similar concerns under the "giftedness" objection to genetic modification. The basic theme that unites these different concerns is that genetic modification treats children as products to be designed, perfected, manipulated, and controlled. Children are no longer viewed as gifts, but as commodities. Jonas[15]and Kass[14] have voiced the concern that various forms of assisted reproduction can have a detrimental affect on how parents view their children. More recently, Michael Sandel has developed the argument that genetic modification is problematic because it represents

...a kind of hyperagency – a Promethean aspiration to remake nature, including human nature, to serve our purposes and satisfy our desires. The problem is not the drift to mechanism but the drive to mastery. And what the drive to mastery misses and may even destroy is an appreciation of the gifted character of human powers and achievements...The problem is not that parents usurp the autonomy of a child they design. The problem lies in the hubris of the designing parents, in their drive to master the mystery of birth [[16] at pg 54, 56].

The problem with genetic enhancement, according to this argument, is that it gives parents too much control over the traits of their children. In another common articulation of this critique, parents and others supporting genetic modification are accused of desiring to "play God", and of designing children to fulfill their own desires.

This argument straightforwardly assumes a strong version of genetic determinism: genetics can be used to control traits only if those traits are strongly determined by genetics in the first instance. But with the knowledge that most traits are not strongly determined by genetics the vision of hordes of parents shaping their children and, in the process, remaking nature loses its cogency. Parents may be able to influence nature but they are surely unable to *master *it. Even if there is, as Sandel suggests, a "drive to mastery," a wide array of limitations – the inherently limited causal relationship between genes and traits, a lack of actual scientific or technological mastery, etc. – strongly suggest that our ability to exert control via genetic modification will *necessarily *fall far short of anything that could be construed as mastery. That genetic modification encourages a "drive to mastery" is not nearly as worrisome once we understand that it is a drive that must inevitably fall far short of anything resembling actual mastery.

Once one sees through the strong deterministic assumptions that buttress the giftedness argument it loses its persuasive power. And once parents realize that they cannot substantially control the genetic composition of their children, or the traits those children will express, they are likely to regard their children just as they have traditionally regarded them, as gifts to be accepted rather than as products to be perfected.

#### 3. Genetic modification and authenticity

The third critique we will consider is the claim that genetic modification undermines the authenticity of a person's accomplishments. The basic idea here is that the genetically modified individual's talents and abilities are no longer his own, that they are the product of the modification. Following this argument leads to the conclusion, for example, that a person with genetically-enhanced musical genius is not really a musical genius after all: he or she is a fake. According to the President's Council on Bioethics:

[T]he naturalness of means matters. It lies not in the fact that the assisting drugs or devices are artifacts, but in the danger of violating or deforming the nature of human agency...In most of our ordinary efforts at self-improvement...we sense the relation between our doings and the resulting improvement, between the means used and the ends sought...In contrast, biotechnical interventions act directly on the human body and mind to bring about their effects on a passive subject [[6] at pg 292].

We set to one side the question of whether this concern about authenticity is appropriately directed at genetic modification, especially when we consider all of the other various biotechnical interventions that directly impact individuals' bodies and minds, and note only that this argument also assumes a strong form of genetic determinism. The authenticity argument claims that the person who benefits from a genetic modification plays no significant role in the development of the desired trait. For that to be the case, a person with a gene for musical ability must remain merely a passive subject in the development of his or her musical aptitude, doing nothing important to realize or to improve that ability.

This is strong determinist assumption indeed. Although a genetically modified person may be a passive subject in the development of certain traits, such as eye color or skin color, he or she must take an active role in the development of most traits in which authenticity might be any concern, such as intelligence, athletic ability, social skills, or musical ability. Although genetic modification may confer an advantage in developing or maximizing a particular trait, the genetically modified individual cannot rely on genes alone. To continue with our now well-worn example of musical ability, even a person born with the ideal genotype must still invest considerable time and effort in the practice, performance, and perfection of his or her craft in order to develop into a world-class musician.

As a final note, it clearly makes no difference whether that person's genetic gift results from random assortment of genes during natural human reproduction or from an artificial process, such as genetic modification. In either case the person will be an active agent and not a passive subject. We don't complain too heartily about the authenticity of elite (and "unmodified") athletes, musicians, or intellectuals because we recognize that their achievements are not solely the result of their favorable genetic profile. Why, then, should we suddenly change our tune when an individual's genes are determined by conscious choice rather than by the "genetic lottery." Just as before genes will represent only one of the causal elements, and often a comparatively trivial one, in the individual's ultimate phenotypic expression. In the absence of strongly determining genes there is no reason why the achievements of the genetically modified individual should not be considered fully authentic.

#### 4. Genetic modification and uniqueness

This fourth and final argument postulates that a particular form of genetic modification, cloning, violates the uniqueness of the cloned person. The President's Council on Bioethics argues that cloning would inherently interfere with the individuality of the cloned person and therefore undermine the formation of his or her personal identity:

Cloning-to-produce-children could create serious problems of identity and individuality...Personal identity is, we would emphasize, a complex and subtle psychological phenomenon, shaped ultimately by the interaction of many diverse factors. But it does seem reasonably clear that cloning would at the very least present a unique and possibly disabling challenge to the formation of individual identity...our genetic uniqueness is an important source of our sense of who we are and how we regard ourselves. It is an emblem of independence and individuality. It endows us with a sense of life as a never-before-enacted possibility [[5] at pg 102–103].

In advancing this argument from uniqueness the President's Council makes two key mistakes.

The scientific mistake is the familiar assumption of strong genetic determinism, with the unsupportable conclusion that two individuals with identical genomes will exhibit identical phenotypic expression. Not so. On the basis of the significant physical and behavioral differences found between identical twins[51], as well as for the multitude of reasons discussed above, it seems a near certainty that even genetically identical clones would exhibit very different traits.

The corresponding philosophical mistake made by the President's Council is to assume that genetic composition bears any significant relationship to a person's own self-identity. Arguably, genetic uniqueness matters very little to most people. Most people develop their sense of self from their life experiences, relationships, values, character traits, interests, and skills [51]. Again, the identical twin case is helpful here: identical twins typically have no difficulty viewing themselves as distinct individuals, despite their genetic similarity. Unless an individual were atypically fixated on genetic characteristics a genetically modified individual, even a genetic clone, would be unlikely to use genetic uniqueness to measure his or her sense of self (see Ishiguro, 2005, for a fictional but illuminating example of how individuality may thrive despite a lack of genetic uniqueness) [52]. Stripping away determinist assumptions makes clear once again that genetic modification, even reproductive cloning, is highly unlikely to deprive human beings of uniqueness or personal identity.

## Conclusion

In this article we have argued that four of the most well-worn objections to genetic modification of human beings – the freedom argument, the giftedness argument, the authenticity argument, and the uniqueness argument – rely heavily on deterministic assumptions. These arguments assume that, despite conclusive evidence to the contrary, most traits are strongly determined by genetics (or that individuals believe, and act as if, this is the case). These deterministic assumptions are demonstrably false. Utilizing such false assumptions to support an argument that genetic modification is inherently objectionable exploits the public's worst fears and perpetuates misunderstandings concerning basic human biology and genetics.

So far, most of the debates about the ethics of genetic modification have stayed largely within the confines of academia and have not had a major or lasting impact on public policy. But the day is coming – we think sooner rather than later – when political leaders will be compelled to make difficult choices concerning genetic modification. Thus, the need for well reasoned scientific policy is increasingly a pressing one. Such policy cannot be built on logical or biological errors and misunderstandings: it should rest on a clear and accurate understanding of the best scientific evidence available. As we have seen, the four critiques of genetic modification examined in this paper are frequently used to portray genetic modification as inherently objectionable and immoral, and they are frequently accompanied by the policy suggestion of a ban on further scientific research and development.

Having demonstrated that these non-consequentialist arguments rely on faulty determinist assumptions, we suggest that the public and scientific policy debate should concern itself instead with alternative arguments that address concerns about risk, safety, social and economic consequences, and justice. These alternative, consequentalist arguments tend to support the view that genetic modification is not inherently immoral but that the morality of genetic modification depends on its implementation and its use by individuals and society, and on the consequences produced therein. While predicting the consequences of genetic modification is a speculative exercise we conclude that it is this approach, one free from foundational misconceptions about the deterministic role of genetics in individual development, that is considerably more likely to bear fruit in developing the effective and sustainable science policies that will be so urgently required in the years to come.

## Competing interests

The author(s) declare that they have no competing interests.

## Authors' contributions

DBR and DBV each participated in the conception and design of the research and the drafting and editing of the manuscript. They have both read and approved the final version of the manuscript.
